# Using Muse: Rapid Mobile Assessment of Brain Performance

**DOI:** 10.3389/fnins.2021.634147

**Published:** 2021-01-28

**Authors:** Olave E. Krigolson, Mathew R. Hammerstrom, Wande Abimbola, Robert Trska, Bruce W. Wright, Kent G. Hecker, Gordon Binsted

**Affiliations:** ^1^Centre for Biomedical Research, University of Victoria, Victoria, BC, Canada; ^2^Division of Medical Sciences, University of Victoria, Victoria, BC, Canada; ^3^Faculty of Veterinary Medicine, University of Calgary, Calgary, AB, Canada; ^4^Faculty of Health and Social Development, University of British Columbia Okanagan, Kelowna, BC, Canada

**Keywords:** EEG, ERP, fatigue, cognitive fatigue, performance, health, mobile EEG

## Abstract

The advent of mobile electroencephalography (mEEG) has created a means for large scale collection of neural data thus affording a deeper insight into cognitive phenomena such as cognitive fatigue. Cognitive fatigue – a neural state that is associated with an increased incidence of errorful performance – is responsible for accidents on a daily basis which at times can cost human lives. To gain better insight into the neural signature of cognitive fatigue in the present study we used mEEG to examine the relationship between perceived cognitive fatigue and human-event related brain potentials (ERPs) and electroencephalographic (EEG) oscillations in a sample of 1,000 people. As a secondary goal, we wanted to further demonstrate the capability of mEEG to accurately measure ERP and EEG data. To accomplish these goals, participants performed a standard visual oddball task on an Apple iPad while EEG data were recorded from a Muse EEG headband. Counter to traditional EEG studies, experimental setup and data collection was completed in less than seven minutes on average. An analysis of our EEG data revealed robust N200 and P300 ERP components and neural oscillations in the delta, theta, alpha, and beta bands. In line with previous findings we observed correlations between ERP components and EEG power and perceived cognitive fatigue. Further, we demonstrate here that a linear combination of ERP and EEG features is a significantly better predictor of perceived cognitive fatigue than any ERP or EEG feature on its own. In sum, our results provide validation of mEEG as a viable tool for research and provide further insight into the impact of cognitive fatigue on the human brain.

## Introduction

Over the past decade there has been a rapid increase in the use of mobile electroencephalography (mEEG) to address a range of research questions that have not been possible to ask with more tradition lab-based electroencephalographic (EEG) systems. For instance, Debener et al. (2012) demonstrated that they could collect EEG data while participants were walking and more recently Scanlon et al. (2020) were able to record event-related potentials (ERPs) while participants were riding a bike. Since its advent, the scientific community has questioned the quality of mEEG data – especially of measurements collected by the growing array of low-cost (less than $1,000) mEEG systems. Countering this uncertainty, in prior work (Krigolson et al., 2017) we demonstrated that it was possible to record mEEG data that was comparable to that acquired by a research grade system. Specifically, we demonstrated that we were able to measure the N200 and P300 ERP components – neural responses associated with the engagement of cognitive control and perceptual processing, respectively – using a Muse EEG headband (Krigolson et al., 2017). Importantly, the ERP results we recorded with the Muse EEG headband that were comparable to the ERPs we recorded with a “research grade” Brain Products ActiChamp system. Validating our work, Fickling et al. (2020) replicated our findings with the Muse EEG headband and also demonstrated that they could measure ERPs with this device. In addition, other research groups have demonstrated similar findings with low-cost EEG systems such as the OpenBCI Cyton (Qiu et al., 2019) and the Emotiv Epoc+ (Kotowski et al., 2019; Mercado-Aguirre et al., 2019).

The capability provided by mEEG to rapidly measure neural responses *in situ* provides a way to study factors that affect brain performance on a large scale. For example, it is well established that cognitive (or mental) fatigue has a negative impact on brain performance (Dinges et al., 1997; Lal and Craig, 2002; Borghini et al., 2014; Hopstaken et al., 2015; Trejo et al., 2015). Indeed, increased cognitive fatigue results in increased errors and accidents while driving (Fletcher et al., 2005), flying (Goode, 2003), operating heavy machinery (Tran et al., 2020), making medical decisions (Cammu and Haentjens, 2012), and a wide range of other areas impossible to list here. Increases in cognitive fatigue are typically associated with extended periods of cognitive effort (Trejo et al., 2005) and/or a lack of sleep (Stuster, 2010): but what specifically causes cognitive fatigue to have a negative impact on performance? The negative consequences of cognitive fatigue have been attributed to reductions in action monitoring (Kecklund and Åkerstedt, 1993; Campagne et al., 2004); attention (Boksem et al., 2005), cognitive control (Mizuno et al., 2011), decision-making (Gaba and Howard, 2002), and error-evaluation (Lorist et al., 2005); all of which add up to and result in reductions in performance. It is important to note that a general consensus on the definition of cognitive fatigue is still not agreed upon (Fonseca et al., 2018) but researchers for the most part agree that cognitive fatigue does differ from sleepiness (Phipps-Nelson et al., 2011). In any event, one of the biggest problems with cognitive fatigue is that it is difficult to accurately assess with self-reporting being particularly problematic (Belz et al., 2004; Baranski, 2007; Schmidt et al., 2009; Aidman et al., 2015).

Given the aforementioned issues with self-assessment of cognitive fatigue, it stands to reason that alternate countermeasure detection methodologies are important to develop and validate. With this in mind, a growing body of research has demonstrated that EEG can be used to detect and measure cognitive fatigue and further that EEG provides a viable way to establish a biomarker(s) for cognitive fatigue (Santamaria and Chiappa, 1987; Wijesuriya et al., 2007; Borghini et al., 2014; Clayton et al., 2015; Scammell et al., 2017; Tran et al., 2020). For example, there seems to be a clear relationship between cognitive fatigue and increased theta power (EEG oscillations between 4 and 7 Hz: Torsvall and Åkerstedt, 1988; Cajochen et al., 1996; Aeschbach et al., 1997; Dumont et al., 1997; Caldwell et al., 2002; Lal and Craig, 2002; Macchi et al., 2002; Strijkstra et al., 2003; Campagne et al., 2004; Lin et al., 2005, 2008; Papadelis et al., 2006; Borghini et al., 2012; Craig et al., 2012; Picot et al., 2012; Trejo et al., 2015; Arnau et al., 2017). Additionally, researchers have also observed relationships between cognitive fatigue and increased alpha power (EEG oscillations between 8 and 12 Hz: Torsvall and Åkerstedt, 1988; Åkerstedt et al., 1991; Kecklund and Åkerstedt, 1993; Cajochen et al., 1995, 1996; Dumont et al., 1997; Tanaka et al., 1997; Schier, 2000; Macchi et al., 2002; Campagne et al., 2004; Eoh et al., 2005; Lin et al., 2005, 2008; Papadelis et al., 2006; Pal et al., 2008; Borghini et al., 2012; Picot et al., 2012; Cao et al., 2014; Gharagozlou et al., 2015). A much smaller subset of studies has also shown relationships between increased delta (EEG oscillations between 1 and 3 Hz: Lal and Craig, 2002) and beta power (EEG oscillations between 13 and 30 Hz). Cognitive fatigue does not only impact the EEG power spectra but has also been shown to impact cortical ERPs. In particular, the amplitude and latency of the P300 ERP component have been shown to be reduced and lengthened, respectively, due to increased cognitive fatigue. Indeed, a number of studies have indicated that cognitive fatigue results in diminished P300 amplitudes (Uetake and Murata, 2000; Kato et al., 2009; Schmidt et al., 2009; Zhao et al., 2012; Käthner et al., 2014; Lamti et al., 2016) and increased P300 latencies (Kaseda et al., 1998; Uetake and Murata, 2000).

Of particular interest here are recent accounts which have used linear combinations of EEG and ERP features to improve prediction of cognitive measures such as fatigue. Specifically, while it is obvious from the above review that individual EEG and ERP features correlate with cognitive fatigue, it stands to reason that one might better be able to predict cognitive fatigue (or other cognitive states) using linear combinations of EEG and ERP features. Supporting this idea, Mathewson et al. (2012) found that a regression model using a linear combination of EEG and ERP features was better able to predict the learning rate of participants playing a video game than when they solely examined the relationship between the EEG and ERP features and learning rate separately. In another study, Broadway et al. (2015) paralleled Mathewson et al.’s finding and found that reading comprehension was more accurately predicted by a regression model that included multiple EEG and ERP features (pre-stimulus alpha power, P1 ERP component asymmetry, and a left-hemisphere N1 ERP component) than by any individual EEG or ERP feature on its own. More recently, Ghosh-Hajra et al. (2016) (see also Fickling et al., 2019) have proposed that ERP amplitudes and latencies can be combined as “brain vital signs” to predict the impact of conditions such as concussion on brain function. In sum, these studies provide evidence that cognitive states such as fatigue are better represented as a combination of EEG and ERP features than by any individual feature in isolation.

As we noted at the outset, mEEG affords an ability to rapidly measure neural data thus it provides a way to directly measure issues with brain performance such as cognitive fatigue. Further, given the quick-setup time and measurement capability afforded by the increasing array of low-cost EEG systems (less than 10 min: see Krigolson et al., 2017 for more detail) it is also possible to test large numbers of people in a short amount of time. Here, we decided to take advantages of the capabilities afforded by mEEG and conduct a large-scale study (*n* = 1,000) of the relationship between EEG/ERP features and perceived cognitive fatigue. In our study participants first completed a quick assessment of perceived cognitive fatigue and then completed a simple visual oddball task on an Apple iPad while EEG data was recorded from a Muse EEG headband. Given that self-reported measured of cognitive have fatigue have been found to be unreliable (e.g., Aidman et al., 2015), we also recorded the amount of hours that each participant had been awake as this also has been shown to be a reliable proxy for cognitive fatigue (Buysse et al., 2003; Dorrian et al., 2011; Vejvoda et al., 2014). As an important experimental constraint to emphasize that EEG assessments can be done quickly and almost anywhere, we ensured that testing sessions took place in under 10 min and we collected data at a wide range of venues (in a shopping mall, in cafeterias, in the work place, and at our university). Our primary hypothesis was that we would see relationships between various EEG (theta and alpha power) and ERP (P300 amplitude and latency) features and behavioral measures of cognitive fatigue in line with previous research (e.g., Tanaka et al., 1997; Schmidt et al., 2009). Additionally, we also sought to provide further evidence that linear multiple regression can be used to combine EEG and ERP features to better predict cognitive states such as fatigue relative to when these relationships between EEG and ERP features and a cognitive state are examined individually (Mathewson et al., 2012; Broadway et al., 2015; Qin et al., 2016; Abiri et al., 2019; Jin et al., 2019). Finally, as a final goal we sought to provide further evidence that low-cost mEEG systems like the Muse are a viable and accurate means for measuring EEG and ERP data.

## Materials and Methods

### Participants

Participants from across the province of British Columbia, Canada (*n* = 1,000; 521 females, age range: 18 to 62) participated in the present experiment. It is important to note that participants with more than 50% of their data discarded (see below) were not included in our analysis and we kept testing until we achieved our *a priori* set sample size of 1,000. Thus, we report here that we included the first 1,000 participants that met this criterion and we tested 1,065 participants to achieve this sample size. Data were collected at the University of Victoria, in two industrial locations^1^, and at the Bay Centre Mall in Victoria, B.C., Canada. Our ethics protocol allowed participants to provide full, partial, or no identification or demographic data; as such, some participants chose to withhold this information. All participants had normal or corrected-to-normal vision, no known neurological impairments and provided informed consent approved by the Human Research Ethics Board at the University of Victoria (HREB: BC17-456). The study followed ethical standards as prescribed in the 1964 Declaration of Helsinki and subsequent revisions.

### Apparatus and Procedure

**FIGURE 1:**
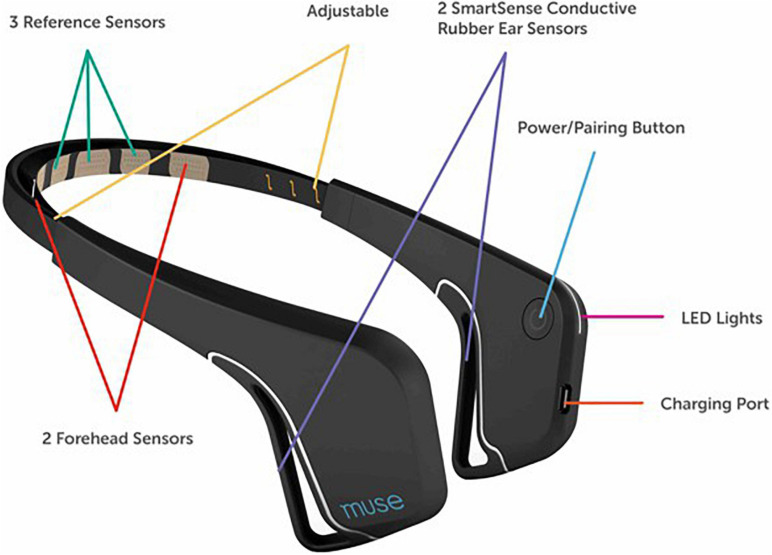
The 2016 Muse EEG system made by InterAxon Inc.

Participants at each testing site completed a standard visual oddball task on an Apple iPad mini (Apple Inc., Cupertino, CA, United States) while EEG data were recorded from a 2016 Muse EEG system (InterAxon Inc., Toronto, ON, Canada: see Figure 1). The visual oddball task and data recording were programed with custom code in the iOS programing environment^2^. Prior to beginning EEG testing, participants self-assessed their perceived level of cognitive fatigue with a modified version of the Swedish Occupational Fatigue Inventory (SOFI-C: Åhsberg et al., 1997) that resulted in a score between 0 (no fatigue) and 5 (very fatigued). We additionally asked participants how many hours they had been awake prior to testing as this has shown to be a predictor of cognitive fatigue (e.g., Dorrian et al., 2011). We also asked participants how many hours they had slept the night before.

During performance of the oddball task participants saw a series of blue (RGB value = [0, 0, 255]) and green (RGB value = [0, 255, 0]) colored circles that appeared for 800 ms in the center of a dark gray background (RGB value = [108, 108, 108]) on the iPad screen. Prior to the onset of the first circle and in between the presentation of subsequent circles a black (RGB value = [0, 0, 0]) fixation cross was presented for 200 to 500 ms, thus, to reduce overall testing time the presentation of the fixation cross also served as the inter-trial interval. Participants were not told that the frequency of the blue and green circles differed: blue circles appeared less frequently (oddball: 30%: mean 30.1 [29.2, 31.0]) than green circles (control: 70%: mean 70.0 [69.1, 70.9]) in a random sequence order. Note, the presentation order of circles was truly random and drawn with replacement; however, the stimulus presentation software did ensure that no more than two infrequent target circles appeared in a row. Participants were instructed to quickly press the bottom left or right corner of the iPad screen with their thumb whenever they saw one of the infrequent target blue circles (henceforth termed: oddball) and to not respond when they saw one of the frequent green circles (henceforth termed: control). The circles were presented for 1,000 ms and the trial ended automatically on oddball trials if participants did not respond. As noted above, there was no inter-trial interval, the next experimental trial began with a fixation cross as soon as the oddball/control circle disappeared. Participants completed four blocks of 50 trials during performance of the oddball task. The total task duration including a signal quality check was seven minutes on average (428 s [344, 512]).

### Data Acquisition

Electroencephalographic data were recorded from a Muse EEG headband (Muse Version: 2016) sampling at 256 Hz (see www.choosemuse.com for full technical specifications). The Muse EEG system has electrodes located analogous to Fpz, AF7, AF8, TP9, and TP10 with electrode Fpz utilized as the reference electrode during recording. Using the Muse SDK, we streamed EEG data via Bluetooth from the Muse EEG system directly to custom iOS software that also presented the experimental stimuli. We did not time synchronize the experimental stimuli with event markers as per a traditional ERP study (Luck, 2014) but instead read the EEG data in with known Bluetooth lag and jitter – which we have already demonstrated still results in a reliable albeit diminished ERP response (Krigolson et al., 2017: see this paper for full details on Bluetooth timing issues and delay/jitter data). Specifically, we “marked” the EEG data at the exact onset time the circles were drawn but because of the Bluetooth lag the EEG samples corresponding to this point in time did not arrive for 40 ms (∼10 samples; ±5 sample of jitter) on average (see Krigolson et al., 2017). It is important to note that this jitter only impacted the initial signal locking between the Muse EEG system and our software and did not change or grow over time. As such, the signal did not vary trial to trial but participant to participant. Signal quality was inferred by examining the variance per second on each EEG channel and data collection began when all channels had a variance per second less than 200 (see Krigolson et al., 2017) for more detail. Finally, our custom software computed the number of trials lost per experimental block in real-time. If a block had more than 50% lost trials, the Muse was adjusted to improve signal quality and the block was repeated. In order to keep testing times within our 10 minute criterion, at most we only repeated one block of trials per participant (if necessary).

### Data Processing and Analysis

Data were processed offline in MATLAB using EEGLAB (Delorme and Makeig, 2004) and custom code^3^. We did not re-reference the continuous EEG data offline as our ERP analysis was focused on the two posterior Muse electrodes (TP9 and TP10) that were referenced appropriately at the time of recording to electrode FPz. Continuous EEG data were filtered with a dual pass Butterworth filter with a passband of 0.1 to 30 Hz then with a 60 Hz notch filter. A preliminary analysis of the data revealed no lateralized effects; further, we wanted to improve the signal-to-noise ratio of the ERP measures (Oken and Chiappa, 1986) so we created a pooled frontal and a pooled posterior virtual electrode by averaging across the frontal (AF7 and AF8) and the posterior (TP9 and TP10) electrodes, respectively. Based on our previous work (Krigolson et al., 2017) our ERP analysis only focused on the new average posterior virtual electrode whereas our EEG analysis via fast Fourier transform (FFT) did examine both the averaged frontal and posterior electrodes. We note that we also chose to not analyze the ERP effects for the frontal ERP channels as re-refencing of the EEG data just mirrors the components given the high correlation between the Muse EEG channels – see https://www.krigolsonlab.com/muse-research.html for exploratory analyses examining this issue that provided the rational for the choices we made here.

#### ERP Analysis

Subsequent to filtering, epochs of data from 200 ms before to 600 ms after stimulus onset (oddball, control) were extracted from the continuous EEG data and were baseline corrected using the 200 ms preceding stimulus onset. An artifact rejection algorithm was then implemented; as a result of this procedure segments that had an absolute difference of more than 60 uV were discarded (on average: 35% [33.5%, 37.5%]). Segments were then averaged for the oddball and control trials for each participant and a difference waveform was constructed by subtracting the average control from the average oddball ERP waveform. Grand average ERPs were generated by averaging all conditional (oddball, control) and difference waveforms for each participant and the peak component latencies were identified (N200: 270 ms; P300: 408 ms). At the participant level, N200 and P300 ERP component amplitudes and latencies were quantified by finding the local minimal (N200: 160 to 380 ms) and local maximal (P300: 215 to 600 ms) voltage amplitudes and latencies with windows around the grand average component peaks.

#### FFT Analysis

Starting again with the raw continuous EEG data for the task, following the filtering process, we divided the entire continuous data into 2,000 ms segments with 1,000 ms overlap. Note, these segments were not separated by condition but instead were representative of the EEG data throughout performance of the oddball task. We then used the same artifact rejection as above to remove segments with an absolute difference of more than 60 uV (on average: 33% [31%, 35%]). We then conducted a FFT using the standard MATLAB function similar to Cohen (Cohen et al., 2008; Cohen, 2014). The FFT was not tapered and the output was normalized. FFT results were standardized and then averaged and power was calculated for the front and back electrodes for the delta (1 to 3 Hz), theta (4 to 7 Hz), alpha (8 to 12 Hz), and beta (13 to 30 Hz) bands for each participant.

#### Statistical Analysis

As we only had a solitary experimental condition and a visual inspection of the confidence intervals on the grand average waveform revealed a distinct ERP response, we did not do any inferential statistics on the ERP components (amplitude and latency) nor EEG power directly. Pearson r correlation values were used to explore to explore the relationship between the ERP and EEG features and the reported behavioral measures (perceived cognitive fatigue, hours awake, and hours asleep). Additionally, stepwise linear regression was used to assess which combination of features was most predictive of perceived cognitive fatigue. The pertinent statistical assumptions for this analysis were tested by examining the distribution of the residuals and also Q–Q plots and all assumptions were met (Tabachnick and Fidell, 2018). An alpha value of 0.05 was assumed for all statistical tests. All descriptive statistics are reported with the mean and the 95% confidence interval.

## Results

**FIGURE 2:**
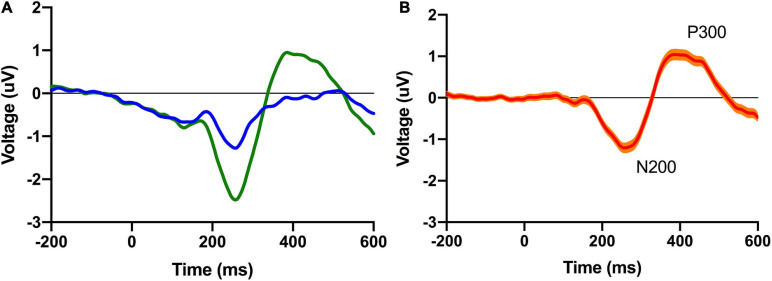
Grand average conditional (left; **A**) and difference (right; **B**) ERP waveforms for the pooled posterior virtual electrode. The 95% confidence interval is plotted on the difference waveform.

**FIGURE 3:**
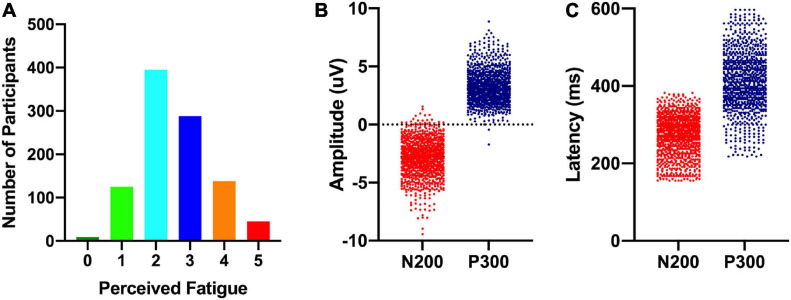
Distribution of perceived cognitive fatigue scores (left; **A**), ERP component amplitudes (middle; **B**), and ERP component latencies (right; **C**).

Participants completed a shortened version of the Perceived Fatigue Scale (mean = 2.6 [2.5 2.7]: see Figure 2) and we additionally recorded self-reported numbers for how long participants were awake before they took part in the experiment (mean = 4.4 h [4.3, 4.6]) and how many hours they had slept the night before (mean = 7.0 [6.9, 7.1]). As noted above, participants also completed a standard visual oddball task on an Apple iPad with EEG data recorded from a Muse EEG headband. An analysis of our EEG data revealed a standard visual ERP response with identifiable N200 and P300 ERP components (see Figure 3). The amplitude (N200: mean = −2.9 uV [−2.8, −3.0]; P300: mean = 3.2 uV [3.1, 3.3]) and latency (N200: mean = 265 ms [260, 270]; P300: mean = 420 ms [409, 431]) were in line with previous work done by our laboratory (see Figure 1; also see Krigolson et al., 2017).

**FIGURE 4:**
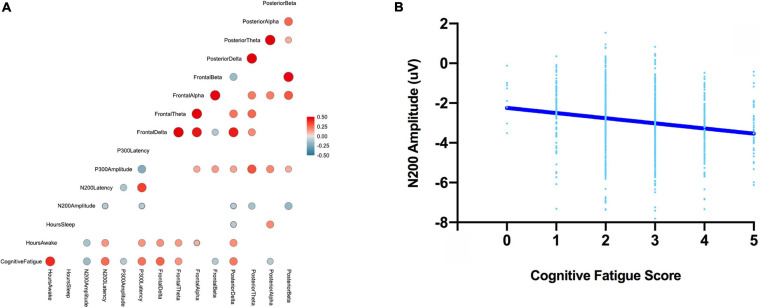
The correlation matrix between all ERP, EEG features, perceived cognitive fatigue scores, hours awake, and hours asleep (left; **A**). Note, all correlations greater than –0.1 and less than 0.1 are not shown. A sample scatter plot for one of the observed relationships – N200 amplitude and perceived cognitive fatigue (right, **B**).

Pearson r correlations were computed between the ERP (amplitude and latency) and EEG (power in the delta, theta, alpha, and beta bands for frontal and posterior electrodes) features and behavioral measures (Table 1). Absolute significant correlations ranged from −0.17 to 0.30 representing small to medium strength relationships between ERP/EEG features and behavioral measures (Cohen, 1988); see Figure 4 for the correlation matrix and a sample representation of one of the relationships that we observed. To examine how linear combinations of our ERP and EEG features predicted perceived cognitive fatigue we used stepwise multiple regression. The results of this analysis revealed a model that predicted perceived cognitive fatigue, *F*(7,992) = 33.1, *p* < 0.001 (*r* = 0.50, *r*^2^ = 0.25, Table 2) that included Frontal Delta, N200 Latency, P300 Latency, Posterior Alpha, N200 Amplitude, Posterior Delta, Posterior Theta, and Frontal Beta.

**TABLE 1:** Pearson *r* correlations between perceived fatigue, hours awake, and hours slept and ERP and EEG features.

	**Perceived fatigue**	**Hours awake**	**Hours slept**
N200 Amplitude	*r* = −0.17***	*r* = −0.14**	*r* = 0.00
	*p* < 0.001	*p* < 0.001	*p* = 0.960
N200 Latency	*r* = 0.25***	*r* = 0.16***	*r* = −0.07*
	*p* < 0.001	*p* < 0.001	*p* = 0.029
P300 Amplitude	*r* = −0.11***	*r* = −0.07*	*r* = 0.07*
	*p* < 0.001	*p* = 0.041	*p* = 0.039
P300 Latency	*r* = 0.24***	*r* = 0.16***	*r* = −0.03
	*p* < 0.001	*p* < 0.001	*p* = 0.309
Frontal Delta	*r* = 0.30***	*r* = 0.17***	*r* = −0.05
	*p* < 0.001	*p* < 0.001	*p* = 0.099
Frontal Theta	*r* = 0.16**	*r* = 0.15***	*r* = −0.01
	*p* < 0.001	*p* < 0.001	*p* = 0.884
Frontal Alpha	*r* = 0.04	*r* = 0.10**	*r* = 0.03
	*p* = 0.266	*p* = 0.003	*p* = 0.283
Frontal Beta	*r* = −0.11**	*r* = −0.05	*r* = 0.01
	*p* = 0.001	*p* = 0.091	*p* = 0.727
Posterior Delta	*r* = 0.22***	*r* = 0.17***	*r* = −0.11**
	*p* < 0.001	*p* < 0.001	*p* = 0.001
Posterior Theta	*r* = −0.02	*r* = 0.07**	*r* = 0.08**
	*p* = 0.557	*p* = 0.020	*p* = 0.015
Posterior Alpha	*r* = −0.11**	*r* = 0.00	*r* = 0.18***
	*p* = 0.001	*p* = 0.996	*p* = 0.000
Posterior Beta	*r* = 0.00	*r* = 0.07*	*r* = 0.01
	*p* = 0.990	*p* = 0.014	*p* = 0.823

*Indicates *p* is between 0.05 and 0.01.**Indicates *p* is between 0.001 and 0.01.***Indicates *p* is less than 0.001.

**TABLE 2:** Regression coefficients for the stepwise regression model that predicts self-reported cognitive fatigue.

	***B***	***SE B***	**β**
(Constant)	0.363	0.223	
Frontal Delta	0.075	0.013	0.188***
N200 Latency	0.005	0.001	0.194***
P300 Latency	0.001	0.000	0.135***
Posterior Alpha	–0.083	0.043	−0.069*
N200 Amplitude	–0.087	0.019	−0.132***
Posterior Delta	0.068	0.015	0.181***
Posterior Theta	–0.159	0.054	−0.126***
Frontal Beta	–0.129	0.060	−0.062*

*Indicates *p* is between 0.05 and 0.01.**Indicates *p* is between 0.001 and 0.01.***Indicates *p* is less than 0.001.

## Discussion

In the present study we found that we could measure robust ERP components and EEG power in a large sample (*n* = 1,000) using a mEEG system and an Apple iPad. To validate the usefulness of mEEG for “real world” data collection we collected the data presented here in a range on environments and in under 7 min per participant on average. In line with the principle aim of this study, we found that the majority of our ERP and EEG measurements were correlated with perceived cognitive fatigue (see Table 1). Also in line with the secondary aim of this study, we also found that a regression model that used a linear combination of ERP and EEG features was a better predictor of perceived cognitive fatigue than any of the individual feature measurements in on its own. In sum, our results demonstrate that mEEG can be used to quickly obtain EEG data from a large number of people providing a viable methodology for potential clinical applications such as concussion assessment (e.g., Fickling et al., 2019) and/or the assessment of mild cognitive impairment (Jackson and Snyder, 2008).

The relationships we found between ERP and EEG measurements and cognitive fatigue parallel previous work. For example, the correlations we observed between P300 amplitude and latency and perceived cognitive fatigue mirror previous findings that showed the same relationships – a decrease in P300 amplitude (Zhao et al., 2012) and an increase in P300 latency (Uetake and Murata, 2000) with increased cognitive fatigue. Supporting this relationship, we observed a parallel relationship (decrease in P300 amplitude, increase in P300 latency) with the hours participants were awake – another proxy for cognitive fatigue (Buysse et al., 2003; Dorrian et al., 2011; Vejvoda et al., 2014). Interestingly, we also observed the same relationship - decreased component amplitude, increased component latency – between the N200 ERP component and perceived cognitive fatigue – a finding that to best of our knowledge is novel. As with the P300, the relationships between N200 amplitude and latency with hours participants had been awake were also present. As such, the N200 amplitude and latency also appear to be potential markers for measuring cognitive fatigue. As to why we observed this effect whereas other have not, we can only speculate that the power of the current study allowed us to detect an effect that was previously masked by relatively lower powered studies (*n* on average < 30, e.g., Zhao et al., 2012). The relationships we observed between cognitive fatigue (and hours awake) and EEG power for the most part were in line with previous findings. Specifically, in line with findings by Lal and Craig (2002) and others we observed an increase in frontal delta and frontal theta EEG power with increased cognitive fatigue. However, counter to previous findings, we observed a decrease (as opposed to an increase) in posterior alpha power with increased cognitive fatigue. We speculate here that this result is related to how we measured EEG power in the present study. In the majority of previous studies, EEG power is measured during a resting state whereas here we measured EEG power during actual task performance. Extending from this, it is well established that alpha power is reduced during task performance, most likely due to an increased focus of visuospatial attention (Sauseng et al., 2005; Foxe and Snyder, 2011; Klimesch, 2012). With this in mind, we suggest that the reduction in alpha power that we observed with increased cognitive fatigue reflects a greater demand on attentional resources to achieve successful task performance when one is tired.

When considered together, it is important to note the overlap between the ERP and EEG features in the present study. More specifically, the ERP components investigated here (N200 and P300) have a spectral representation in the delta and theta range (Güntekin and Başar, 2010) and thus might be expected to a common correlated outcome such as perceived cognitive fatigue. With that said, our results show independent contributions of the stimulus locked ERP components and the cross experiment spectral power measures that we computed thus suggesting that there is additional information in stimulus generated non-phase locked elements and thus the ERP and EEG signals do not necessarily reflect the same underlying processes, at least in our experiment (see also Sauseng et al., 2007).

Here we also demonstrated that a linear combination of ERP and EEG features was a better predictor of perceived cognitive fatigue than any individual feature on its own. Indeed, this result is in line with previous work (Mathewson et al., 2012; Broadway et al., 2015; Qin et al., 2016; Abiri et al., 2019; Jin et al., 2019) and highlights that the relationship between complex cognitive constructs such as perceived cognitive fatigue and neural measures such as EEG and ERP responses is better explained using a combination of neural features. For instance, Wang et al. (2012) found that a linear combination of ERP components resulted in better classification of categories of visual objects. In a similar vein, Mathewson et al. (2012) found that a combination of EEG and ERP features was a better predictor of individual learning rate than any of these features in isolation. Indeed, it makes sense that adding additional neural features to a regression model would improve prediction as logically complex phenomena such as cognitive fatigue would impact the entire time course of an ERP waveform or multiple oscillatory frequencies. Further, given that ERP components and EEG frequencies are thought to originate from different regions of the cortex it makes sense that the ERP/EEG measures (and neural regions) are individually differentially impacted by cognitive fatigue and thus combining ERP/EEG features improves prediction. Of course, it is important to use proper statistical methods to ensure overfitting does not occur (Bartlett et al., 2020).

Perhaps the most important point made with the current data is that the advent of mEEG and its validation (see Krigolson et al., 2017) provides a capability to quickly collect EEG data from a number of people. Given recent claims that ERP components can be used potentially for clinical diagnosis of concussion and other clinical conditions (Ghosh-Hajra et al., 2016; Fickling et al., 2019), mass testing with mEEG will allow for the actual computation of population norms for EEG data such as those that already exist for heart rate and blood pressure thus making condition diagnosis and tracking via EEG a viable possibility. Further, given the rapid testing time that is possible with mEEG – less than seven minutes in the present study – and the ease of use of this technology, medical screening via EEG could be done by technicians in a manner akin to how blood samples are presently taken thus alleviating burden on the healthcare system. Finally, mEEG allows data collection in almost any environment – Debener et al. (2012) were able to collect mEEG data outside while someone was walking as an example.

It is important to note than mEEG is not without its problems. Data quality for one, is not as good as a research grade EEG system (Radüntz, 2018). Further, electrodes tend to be placed in non-standard positions for observing “classic” ERP components and EEG oscillations (Krigolson et al., 2017). Perhaps the greatest problem with the approach we used here to collect event-locked EEG data, and also with the use of EEG systems that rely on Bluetooth technology in general, is the known lag and jitter associated with Bluetooth data transmission. The collection of stimulus locked EEG data (i.e., event-related potentials) is typically done with precise event-marking with a goal of less than one millisecond of lag and a very small amount of jitter between a given event and when the EEG data is “marked” for the onset of that event (Luck, 2014). We were unable to use a traditional method to insert EEG event-markers (e.g., a voltage sent on a USB or parallel cable from a stimulus computer) into the Muse EEG data stream with our current experimental setup. The data presented here suffers from both lag and jitter due to Bluetooth transmission. As a result, our stimulus locked ERPs are shifted temporally due to lag and reduced in amplitude due to jitter. Importantly, this is a key issue that is problematic with mEEG technology reliant upon Bluetooth (and to some extent wireless) data transmission – the attenuation of event-locked responses due to temporally jittered event markers. There are other issues regarding Bluetooth data transmission in mEEG experiments that need to be considered. For instance, Bluetooth interference between other Bluetooth devices and/or interference from wireless transmitters at a testing sight might disrupt the transmission of EEG data (Tshiluna et al., 2016). There are potential technological developments that might reduce or remove the impact of Bluetooth data transmission on the EEG signal. For example, Cognionics mobile EEG systems use radio pulses to provide accurate temporal marking of event-locked EEG responses^4^. Another potential solution would be to implement a software solution to assess Bluetooth lag (and temporal jitter) by sending a ping to a mEEG device and measuring the signal return time. Then, in principle, the lag could be accounted for and “correctly timed” EEG event-markers could be sent to the device to compensate for Bluetooth lag and jitter. With that said, it is worth pointing out that even with attenuation, we were still able to see clearly defined ERP responses in the present experiment. Thus, in spite of the issues associated with Bluetooth data transmission, our data demonstrate that mEEG has the capability to expand the use of EEG as a tool in both clinical and research settings.

## Conclusion

In the present experiment we demonstrated in a large sample (*n* = 1,000) that we could quickly and accurately measure ERP and EEG data. Further, we replicated previous work showing relationships between ERP and ERP features and perceived cognitive fatigue and also demonstrated that a linear combination of ERP and EEG features was a better predictor of perceived cognitive fatigue than any one ERP or EEG feature on its own. Finally, our work here affirms the validity of mEEG as a means for measuring brain health and performance in real world environments.

## Data Availability Statement

The raw data supporting the conclusions of this article will be made available by the authors, without undue reservation.

## Ethics Statement

The studies involving human participants were reviewed and approved by Human Research Ethics Board at the University of Victoria. The patients/participants provided their written informed consent to participate in this study.

## Author Contributions

OK spearheaded the research project, managed all data collection sessions, oversaw data analysis, and wrote the manuscript. MH completed data collection in some of the research locations, data analysis, and aided in the writing of the manuscript. WA completed data collection in the majority of the research locations and aided in data analysis. RT completed data collection in the majority of the research locations and aided in data analysis. BW was a senior author and helped with conceptualization, experimental design, and writing. KH aided in data and statistical analysis and writing. GB was helped with conceptualization, experimental design, and writing. All authors contributed to the article and approved the submitted version.

## Conflict of Interest

The authors declare that the research was conducted in the absence of any commercial or financial relationships that could be construed as a potential conflict of interest.

## Footnotes

1 The sites were a mine and a hospital. Our ethics protocol and the sites tested prohibit us from disclosing identity or location but did allow us to use the data for this manuscript.

2 iOS source code is available upon request. A MATLAB version of this code is provided here (https://www.krigolsonlab.com/working-with-muse.html).

3 https://github.com/Neuro-Tools

4 www.cgxsystems.com
